# Frequency and severity of prehospital obstetric events encountered by emergency medical services in the United States

**DOI:** 10.1186/s12884-021-04129-1

**Published:** 2021-09-24

**Authors:** Rebecca E. Cash, Robert A. Swor, Margaret Samuels-Kalow, David Eisenbrey, Anjali J. Kaimal, Carlos A. Camargo

**Affiliations:** 1 Department of Emergency Medicine, Massachusetts General Hospital, Harvard Medical School, Boston, MA USA; 2 Department of Emergency Medicine, Oakland University William Beaumont School of Medicine, Royal Oak, MI USA; 3 Department of Emergency Medicine, McLaren Flint Hospital, Flint, MI USA; 4 Division of Maternal Fetal Medicine, Department of Obstetrics and Gynecology, Massachusetts General Hospital, Harvard Medical School, Boston, MA USA

**Keywords:** Emergency medical services, Prehospital, Obstetrics, Childbirth

## Abstract

**Background:**

Prehospital obstetric events encountered by emergency medical services (EMS) can be high-risk patient presentations for which suboptimal care can cause substantial morbidity and mortality. The frequency of prehospital obstetric events is unclear because existing descriptions have reported obstetric and gynecological conditions together, without delineating specific patient presentations. Our objective was to identify the types, frequency, and acuity of prehospital obstetric events treated by EMS personnel in the US.

**Methods:**

We conducted a cross-sectional analysis of EMS patient care records in the 2018 National EMS Information System dataset (n=22,532,890). We focused on EMS activations (i.e., calls for service) for an emergency scene response for patients aged 12-50 years with evidence of an obstetric event. Type of obstetric event was determined by examining patient symptoms, the treating EMS provider’s impression (i.e., field diagnosis), and procedures performed. High patient acuity was ascertained by EMS documentation of patient status and application of the modified early obstetric warning system (MEOWS) criteria, with concordance assessed using Cohen’s kappa. Descriptive statistics were calculated to describe the primary symptoms, impressions, and frequency of each type of obstetric event among these activations.

**Results:**

A total of 107,771 (0.6%) of EMS emergency activations were identified as involving an obstetric event. The most common presentation was early or threatened labor (15%). Abdominal complaints, including pain and other digestive/abdomen signs and symptoms, was the most common primary symptom (29%) and primary impression (18%). We identified 3,489 (3%) out-of-hospital deliveries, of which 1,504 were preterm. Overall, EMS providers documented 34% of patients as being high acuity, similar to the MEOWS criteria (35%); however, there were high rates of missing data for EMS documented acuity (19%), poor concordance between the two measures (Cohen’s kappa=0.12), and acuity differences for specific conditions (e.g., high acuity of non-cephalic presentations, 77% in EMS documentation versus 53% identified by MEOWS).

**Conclusion:**

Prehospital obstetric events were infrequently encountered by EMS personnel, and about one-third were high acuity. Additional work to understand the epidemiology and clinical care of these patients by EMS would help to optimize prehospital care and outcomes.

**Supplementary Information:**

The online version contains supplementary material available at 10.1186/s12884-021-04129-1.

## Background

Thousands of mothers in the US give birth outside of a healthcare setting or experience an obstetric event each year [1], often resulting in treatment by emergency medical services (EMS). However, little is known regarding prehospital obstetric events in the US. Current national estimates of the frequency of these events do not differentiate between gynecological and obstetric complaints [2–4], nor are the specific conditions treated most commonly by EMS personnel known. The paucity of data presents a challenge for improving the education, training, and clinical protocols of EMS personnel. Similarly, there are no evidence-based guidelines covering prehospital obstetric care beyond recent efforts to address maternal resuscitation during cardiac arrest [5].

Evidence from other countries suggests that obstetric events, including events like unplanned out-of-hospital delivery or post-partum hemorrhage, are low frequency but potentially high-risk patient presentations. Between 0.5-1% of all emergency EMS calls are estimated to be an EMS-attended delivery [6, 7]. Unplanned out-of-hospital deliveries are associated with increased risk of major complications such as postpartum hemorrhage, neonatal intensive care admission, and maternal or neonatal death [8–13]. Because of limited exposure to these patients, many EMS personnel feel unprepared or even fear having to manage the prehospital care of a pregnant patient [14]. Furthermore, a better understanding of the acuity of patients presenting with obstetric conditions would enable improved EMS planning and training.

The goal of this study was to provide a current description of prehospital obstetric conditions and emergencies treated by EMS personnel. Our objective was to identify the types, frequency, and acuity of obstetric events treated by EMS in the US. Elucidating the types of patients and situations EMS personnel encounter is the first step to understanding how to improve obstetric care in the prehospital setting.

## Methods

### Study design, setting, & participants

This was a cross-sectional analysis of EMS patient care records in the National EMS Information System (NEMSIS) Public Release Research Dataset Version 3.4 [15]. NEMSIS, a collaboration with the National Highway Traffic Safety Administration’s Office of EMS and the University of Utah, is the largest repository of EMS patient care records in the United States, containing records for nearly 30 million activations (i.e., calls for service) each year from over 10,000 EMS agencies in 47 states and territories [15]. EMS agencies and states submit a standardized set of national data elements for each record, which are then de-identified and made publicly available for research purposes.

For this study, we used EMS activations for an emergency scene response (i.e., after a 9-1-1 call) with patient involvement from January 1, 2018 to December 31, 2018. We included activations for patients aged 12-50 years with a documented dispatch reason, symptom, impression, or procedure indicating an obstetric complaint, including out-of-hospital delivery. Because only patient gender, rather than biological sex, was recorded, we did not place restriction on the documented gender so as to not exclude transgender men. We excluded activations for a non-emergency response (e.g., interfacility or non-emergency medical transport); where the response was cancelled without patient contact or no patient was found on scene; if the patient was outside of the specified age range (including if age was missing); or if the record had no indication of an obstetric complaint or pregnancy.

The MassGeneral Brigham institutional review board reviewed this study and deemed it non-human subjects research due to the de-identified nature of the publicly available data.

### Measures

We identified an EMS emergency activation involving an obstetric complaint based on one or more of the following: dispatch reason; primary and secondary symptoms documented by the treating EMS provider; the EMS provider’s primary and secondary impressions (i.e., field diagnosis) of the patient’s complaint; procedures performed by EMS; and the EMS provider’s documentation of the clinical protocol under which they provided care (Table S1). Dispatch reason was a standardized list of general complaints [15], from which we selected “2301057 – Pregnancy/childbirth/miscarriage.” Patient symptoms and provider impressions were based on a subset of International Classification of Diseases, Tenth Revision, Clinical Modification (ICD-10-CM) codes [16], for which we selected codes beginning with: O, P, Z32-Z39, and Z3A. Neonatal codes were included because care of the mother and baby are frequently included in the same EMS record. Procedures were based on the Standardized Nomenclature of Medicine Clinical Terms (SNOMED CT) [17], for which we selected 26 procedures relating to out-of-hospital delivery and care of the newborn. For the clinical protocol used during the activation, we selected five protocols relating to pregnancy, childbirth, and neonatal resuscitation from 112 total protocols.

We examined the primary symptom and primary impression of the emergency activations identified as being for an obstetric complaint or pregnant patient. We used the Agency for Healthcare Research and Quality (AHRQ) Clinical Classifications Software Refined (CCSR) [18] categories to group the ICD-10-CM codes for these two variables into meaningful categories. We then further examined the frequency of 14 specific types of obstetric events treated by EMS, also based on the AHRQ CCSR categories. The categories included: early, first, or unspecified trimester hemorrhage; early or threatened labor; eclampsia, preeclampsia, and hypertensive conditions; ectopic or molar pregnancy and complications; intra- and post-partum hemorrhage; malposition, disproportion, or other labor complications; maternal cardiac arrest (any etiology); multiple gestation delivery; non-cephalic presentation; nuchal cord; out-of-hospital birth/delivery; preterm delivery; prolapsed cord; and spontaneous or induced abortion and complications. Table S2 lists the ICD-10 codes associated with each category.

As a secondary outcome, we examined the acuity of the patient as documented by the treating EMS provider in two ways. First, we defined a high acuity patient as one where the EMS provider documented the initial and/or final patient acuity as “critical,” “emergent,” or “dead without resuscitation efforts.” The definitions of patient acuity in NEMSIS are based on the National Highway Traffic Safety Administration’s National EMS Core Content [19]. However, EMS provider documented patient acuity is subjective since the decision is based on their own clinical judgment rather than a standardized measure. Activations where acuity was documented as not applicable or not recorded were considered missing and not included in the denominator. Second, because of the subjective and potentially inconsistent nature of the EMS provider documented patient acuity as well as high rate of missingness, we used the modified early obstetric warning system (MEOWS) [20, 21] to determine an objective measurement of patient acuity (Table S3). MEOWS consists of 8 physiological parameters with “red” and “yellow” alert criteria that was designed to be used to trigger evaluation of obstetric patients who may be at high risk of clinical deterioration and impending critical illness [20, 21]. We did not include the parameters for diastolic blood pressure and patient temperature because these were not available in the dataset. High acuity was defined as meeting one red or two yellow alert conditions, in line with the recommendations for its use for early recognition of high acuity obstetric patients. The MEOWS scoring was calculated for all activations where data were available for at least 4 of the 6 components.

### Data analysis

Descriptive statistics were calculated to describe the frequency of each type of obstetric event and the primary symptoms and impressions among these activations. Among each type of obstetric event, we also examined the proportion considered high acuity. Available case analysis was used to handle missing data. Concordance between the two measures of patient acuity was determined using Cohen’s kappa statistic. Analysis was conducted using Stata IC 15.1 (StataCorp, College Station, TX).

## Results

**Fig. 1:**
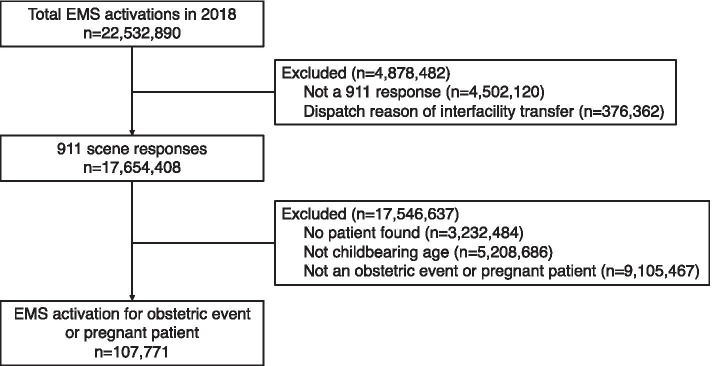
Study flowchart. Abbreviation: EMS, emergency medical services

In 2018, there were a total of 22,532,890 EMS activations included in the NEMSIS dataset, of which 17,654,408 were an emergency scene response (Fig. 1). After applying the additional exclusion criteria (Fig. 1 and Table S1), we identified a total of 107,771 activations for an obstetric complaint or pregnant patient, representing 0.6% of all EMS emergency activations.

For all obstetric activations, the three most common primary symptoms (Table 1) were non-specific and included abdominal complaints, including pain and other digestive/abdomen signs and symptoms (n=29,350, 29%); other general signs and symptoms (n=12,901, 13%); and other specified female genital disorders (n=9,802, 10%). The most common primary impression (Table 2) was also abdominal complaints, including pain and other digestive/abdomen signs and symptoms (n=18,222, 18%), followed by other specified complications in pregnancy (n=15,350, 15%) and malposition, disproportion, or other labor complications (n=13,743, 13%).

**Table 1:** Primary symptom^a^ documented by treating provider for EMS emergency activations for an obstetric event (n=107,771)

Category	Emergency Obstetric Activations, n (%)
Abdominal pain and other digestive/abdomen signs and symptoms	29,350 (29)
Other general signs and symptoms	12,901 (13)
Other specified female genital disorders	9,802 (10)
Early or threatened labor	9,539 (10)
Uncomplicated pregnancy, delivery or puerperium	8,186 (8)
Circulatory signs and symptoms	4,107 (4)
Nausea and vomiting	3,523 (4)
Malaise and fatigue	2,052 (2)
Other specified complications in pregnancy	1,879 (2)
Medical examination/evaluation	1,810 (2)
Musculoskeletal pain, not low back pain	1,697 (2)
Malposition, disproportion or other labor complications	1,471 (1)
Symptoms of mental and substance use conditions	1,429 (1)
Nervous system signs and symptoms	1,052 (1)
Early, first or unspecified trimester hemorrhage	866 (1)
Other obstetric conditions	2,504 (3)
Other non-obstetric conditions^b^	7,369 (8)

Note: Percentages may not total to 100% due to rounding

Abbreviations: EMS, emergency medical services

^a^Primary symptom (eSituation.09) grouped by the Agency for Healthcare Research and Quality Clinical Classifications Software Refined (CCSR) for International Classification of Diseases, 10th Revision, Clinical Modification (ICD-10-CM) Diagnoses version 2020.3 categories [18]. Default CCSR categories were used except for “Z37” codes (classified instead as maternal outcome of delivery)

^b^Other includes 107 categories under 1% each

**Table 2:** Primary impression^a^ documented by treating provider for EMS emergency activations for an obstetric event (n=107,771)

Category	Emergency Obstetric Activations, n (%)
Abdominal pain and other digestive/abdomen signs and symptoms	18,222 (18)
Other specified complications in pregnancy	15,350 (15)
Malposition, disproportion or other labor complications	13,743 (13)
Uncomplicated pregnancy, delivery or puerperium	12,336 (12)
Other specified female genital disorders	9,582 (9)
Early or threatened labor	6,869 (7)
Complications specified during childbirth	6,003 (6)
Spontaneous abortion and complications	2,689 (3)
Malaise and fatigue	1,506 (1)
Nervous system pain and pain syndromes	1,354 (1)
Nausea and vomiting	1,118 (1)
Other obstetric conditions	2,276 (2)
Other non-obstetric conditions^b^	12,235 (12)

Note: Percentages may not total to 100% due to rounding

Abbreviations: EMS, emergency medical services

^a^Primary impression (eSituation.11) grouped by the Agency for Healthcare Research and Quality Clinical Classifications Software Refined (CCSR) for International Classification of Diseases, 10th Revision, Clinical Modification (ICD-10-CM) Diagnoses version 2020.3 categories [18]. Default CCSR categories were used except for “Z37” codes (classified instead as maternal outcome of delivery)

^b^Other includes 143 categories under 1% each

Of the 14 pre-specified conditions of interest based on the AHRQ CCSR categories, the most common type of obstetric complaint was early or threatened labor (Table 3; n=15,936, 15% of all obstetric activations). We identified 3,489 (3%) out-of-hospital deliveries, of which 1,504 were a preterm delivery. There were 1,395 (1%) activations for hemorrhage during pregnancy and 629 (0.6%) activations for hemorrhage during or after delivery. Maternal cardiac arrest was documented in 25 (<0.1%) activations. There was a very low frequency of multiple gestation delivery and nuchal cord (<10 events each).

**Table 3:** Frequency of emergency medical services EMS emergency activations for selected obstetric events (n=107,771)^a^

Category	Emergency Obstetric Activations, n (%)
Early or threatened labor	15,936 (15)
Malposition, disproportion, or other labor complications	15,433 (14)
Prolapsed cord	21 (<0.1)
Non-cephalic presentation	37 (<0.1)
Spontaneous or induced abortion and complications	4,001 (4)
Out-of-hospital birth/delivery	3,489 (3)
Preterm delivery	1,504 (1)
Early, first or unspecified trimester hemorrhage	1,395 (1)
Eclampsia, preeclampsia, and hypertensive conditions	1,000 (1)
Intra- and post-partum hemorrhage	629 (0.6)
Ectopic or molar pregnancy and complications	332 (0.3)
Maternal cardiac arrest, any etiology	25 (<0.1)

Note: Percentages may not total to 100% due to rounding.

Abbreviations: EMS, emergency medical services

^a^Identification based on any symptom, impression, or procedure documented. Each EMS activation could be included under multiple categories (e.g., out-of-hospital delivery with post-partum hemorrhage). Multiple gestation delivery and nuchal cord were also examined but not presented due to being <10 events each

The EMS provider’s documentation of acuity was missing or not recorded in a substantial number of observations (19% overall; Table 4). Missing data for MEOWS was much lower at 3% overall, but for 28% of maternal cardiac arrest cases we could not calculate a MEOWS score because of missing data (e.g., missing vital signs). The proportion missing for each condition is reported in Table 4, ranging from 4% to 30% missing for EMS provider documented acuity. However, among activations with an EMS provider documented acuity (n=87,538), a total of 30,131 (34%) were considered high acuity. The conditions with the highest proportion of activations that were high acuity were non-cephalic presentation (77%), maternal cardiac arrest (63%), and preterm delivery (59%). Similarly, using the MEOWS criteria for high acuity, 35% of obstetric activations were considered high acuity. Concordance between the two measures of acuity was relatively poor, with agreement of 60% and a Cohen’s kappa statistic of 0.12. The highest acuity conditions based on the MEOWS criteria were eclampsia, preeclampsia, and hypertensive conditions (70%); maternal cardiac arrest (67%); and non-cephalic presentation (53%). For situations where the fetus and newborn’s clinical condition was at higher risk than the mother, acuity based on the MEOWS criteria was substantially lower than the EMS provider documentation – e.g., 77% of non-cephalic presentations were considered high acuity by the EMS crew versus 53% identified by MEOWS criteria.

**Table 4:** Proportion of emergency activations for obstetric events treated by EMS that were considered high acuity and missing data on patient acuity

Category	Per EMS Provider^a^		Per MEOWS^b^	
	n (row %)	***Missing,*** ***n (row %)***	n (row %)	***Missing,*** ***n (row %)***
Overall	30,131 (34)	*20,233 (19)*	36,761 (35)	*3,044 (3)*
Early or threatened labor	5,437 (42)	*3,124 (20)*	5,767 (37)	*413 (3)*
Malposition, disproportion, or other labor complications	4,873 (39)	*2,896 (19)*	5,040 (33)	*380 (2)*
Prolapsed cord	8 (53)	*6 (29)*	10 (48)	*0*
Non-cephalic presentation	20 (77)	*11 (30)*	18 (53)	*3 (8)*
Spontaneous or induced abortion and complications	1,312 (41)	*779 (19)*	1,493 (39)	*189 (5)*
Out-of-hospital birth/delivery	1,532 (54)	*655 (19)*	1,499 (45)	*129 (4)*
Preterm delivery	669 (59)	*363 (24)*	634 (44)	*68 (5)*
Early, first or unspecified trimester hemorrhage	426 (41)	*363 (26)*	524 (39)	*35 (3)*
Eclampsia, preeclampsia, and hypertensive conditions	431 (53)	*191 (19)*	687 (70)	*17 (2)*
Intra- and post-partum hemorrhage	281 (55)	*114 (18)*	263 (44)	*27 (4)*
Ectopic or molar pregnancy and complications	142 (47)	*31 (9)*	122 (38)	*10 (3)*
Maternal cardiac arrest, any etiology	15 (63)	*1 (4)*	12 (67)	*7 (28)*

Note: Percentages are per row, not column, and do not total to 100%

Abbreviations: EMS, emergency medical services; MEOWS, modified early obstetric warning system

^a^A total of 87,538 (81%) activations had an EMS provider documented patient acuity. Displayed is the percent of each category documented as having an initial (eSituation.13) or final (eDisposition.19) patient acuity of “critical”, “emergent”, or “dead without resuscitation efforts.” “Not applicable/not recorded” was considered missing and not included in the denominator. Frequency and percentage of missing acuity for each category is reported separately

^b^The MEOWS scoring was calculated for activations where at data were available for least 4 of the 6 components (n=104,727, 97% included). Displayed is the percent of each category considered as high acuity (≥1 red or ≥2 yellow alert conditions). Frequency and percentage of missing for each category is reported separately

## Discussion

In this national study of EMS patient care records, we found that 0.6% of EMS emergency activations in the US were for an obstetric complaint or pregnant patient. Of these activations, the most common symptoms and field impressions were for non-specific conditions. A small proportion involved an EMS-attended out-of-hospital delivery, and about one-third of activations were of high patient acuity. The appropriate triage, care, and transportation of obstetric patients by trained EMS personnel can result in improved outcomes [22–25]. However, lack of exposure to obstetric patients, and minimal education requirements covering obstetric care [26, 27], indicates the need for development of more robust training and evidence-based guidelines.

We were able to identify nearly 3,500 activations for out-of-hospital delivery, far more than in prior work focusing on a single state, hospital system, or EMS agency [6, 11, 28]. As most unplanned at-home births would likely trigger activation of EMS, this number is plausible and consistent with the approximately 3,600 unintended home births in 2018 with a non-physician or non-midwife attendant [1]. Additionally, the overall proportion of EMS emergency activations for an obstetric event (0.6%) was similar to estimates from other counties [6, 7].

Definitive diagnosis is not possible in the prehospital setting; thus, we have relied on the EMS field impressions to classify patients. Concordance of paramedic and physician diagnosis is generally moderate to good (>50% to 100% agreement) but varies by condition and setting [29–32]. No prior studies have examined this specifically for obstetric conditions. Estimates in this study for conditions that are less obvious than out-of-hospital delivery, such as preeclampsia or ectopic pregnancy, may not represent true prevalence. Linkages between prehospital records and hospital or emergency department discharge diagnoses would provide a better estimate, but such linkages and data sharing are not currently implemented on a wide scale in the US.

Previous work has applied early warning scores as an objective measure of patient acuity in the prehospital setting [33, 34], but not an obstetric-specific measure. Because a lack of exposure to obstetric patients could lead to fear in treating complex presentations, it was unclear if EMS providers would perceive these patients as higher acuity than they might actually be, leading to downstream changes in care, such as over-triage. Alternatively, under-triage, due to a lack clinical sophistication regarding obstetric complaints, also would have considerable implications for patient care. Finally, patient acuity was commonly not documented by the treating EMS provider leading to high rates of missing data for this variable.

We found substantial discrepancies and poor concordance between the subjective EMS provider documented acuity and objective acuity based on the MEOWS criteria. For many conditions, a larger proportion of patients were documented as high acuity by EMS providers’ perceptions than by MEOWS criteria, especially when the increased risk was for the fetus or newborn rather than mother – e.g., non-cephalic presentation or preterm delivery – likely because of the focus of MEOWS on maternal status. EMS providers may be missing women at risk for maternal complications identified by MEOWS because they are overly focused on fetal status. We encourage future work to develop guidelines for prehospital triage of obstetric patients, incorporating risks to both mother and fetus, so that patients are directed to the most appropriate care.

The NEMSIS dataset, though a convenience sample, is the largest repository of EMS patient care records and, thereby, provides a large enough sample to identify rare patient presentations [15]. However, this study has several limitations. NEMSIS records do not allow for tracking of patients across time or events, and multiple records could be generated for a single event. Accordingly, the unit of analysis is EMS activation rather than a unique patient or event. As with all administrative data, variations in documentation standards and quality present a potential for misclassification. We may have under- or overestimated the frequency of obstetric events due to incorrect or inaccurate documentation. Other data fields, such as the EMS provider’s narrative report of the event, are not collected. There was a substantial amount of missing data for EMS provider documented patient acuity, with 19% of observations missing overall (range of 4-30% per condition). However, we also examined a second, objective measure (MEOWS) for which 97% of activations had data for ≥4 of the 6 components available. In this study, we focused specifically on identifying the types and frequency of prehospital obstetric events treated by EMS personnel. Further work to describe patient or clinical characteristics is planned.

## Conclusions

Prehospital obstetric events are infrequent in the US, suggesting that EMS personnel may only encounter and treat a few of these patients per year. We encourage additional studies examining the epidemiology and clinical care provided during these encounters and efforts to develop evidence-based guidelines to improve prehospital obstetric care.

## Supplementary Information

**Additional file 1: Table S1**. Identification of EMS 911 activations for a potential obstetric event in patients of childbearing age (12-50 years).

**Additional file 2: Table S2**. Definition of the 14 pre-specified obstetric conditions and events.

**Additional file 3: Table S3**. Scoring for the modified early obstetric warning system (MEOWS).

## Data Availability

The data that support the findings of this study are available from the National Emergency Medical Services Information System Technical Assistance Center (https://nemsis.org) but restrictions apply to the availability of these data, which were used under license for the current study, and so are not publicly available. Data are however available from the authors upon reasonable request and with permission of the National Emergency Medical Services Information System Technical Assistance Center.

## Abbreviations

AHRQ: Agency for Healthcare Research and Quality
CCSR: Clinical Classifications Software Refined
EMS: Emergency medical services
ICD-10-CM: International Classification of Diseases, Tenth Revision, Clinical Modification
MEOWS: Modified early obstetric warning system
NEMSIS: National Emergency Medical Services Information System
SNOMED CT: Standardized Nomenclature of Medicine Clinical Terms
US: United States

## References

[1] United States Department of Health and Human Services (US DHHS), Centers for Disease Control and Prevention (CDC), National Center for Health Statistics (NCHS), Division of Vital Statistics. Natality public-use data 2016-2018, on CDC WONDER Online Database 2019 [Available from: http://wonder.cdc.gov/natality-expanded-current.html. Accessed 3 Jan 2020.

[2] Mueller LR, Donnelly JP, Jacobson KE, Carlson JN, Mann NC, Wang HE (2016). National characteristics of emergency medical services in frontier and remote areas. Prehosp Emerg Care.

[3] Duong HV, Herrera LN, Moore JX, Donnelly J, Jacobson KE, Carlson JN (2018). National characteristics of emergency medical services responses for older adults in the United States. Prehosp Emerg Care.

[4] Wang HE, Mann NC, Jacobson KE, Ms MD, Mears G, Smyrski K (2013). National characteristics of emergency medical services responses in the United States. Prehosp Emerg Care.

[5] Panchal AR, Bartos JA, Cabañas JG, Donnino MW, Drennan IR, Hirsch KG (2020). Part 3: Adult Basic and Advanced Life Support: 2020 American Heart Association Guidelines for Cardiopulmonary Resuscitation and Emergency Cardiovascular Care. Circulation.

[6] McLelland GE, Morgans AE, McKenna LG (2014). Involvement of emergency medical services at unplanned births before arrival to hospital: A structured review. Emerg Med J.

[7] Flanagan B, Lord B, Barnes M (2017). Is unplanned out-of-hospital birth managed by paramedics 'infrequent', 'normal' and 'uncomplicated'?. BMC Pregnancy Childb.

[8] McLelland G, McKenna L, Morgans A, Smith K (2018). Epidemiology of unplanned out-of-hospital births attended by paramedics. BMC Pregnancy Childb.

[9] Rodie VA, Thomson AJ, Norman JE (2002). Accidental out-of-hospital deliveries: An obstetric and neonatal case control study. Acta Obstet Gynecol Scand.

[10] Hadar A, Rabinovich A, Sheiner E, Landau D, Hallak M, Mazor M (2005). Obstetric characteristics and neonatal outcome of unplanned out-of-hospital term deliveries: a prospective, case-control study. J Reprod Med.

[11] Moscovitz HC, Magriples U, Keissling M, Schriver JA (2000). Care and outcome of out-of-hospital deliveries. Acad Emerg Med.

[12] Engjom HM, Morken NH, Hoydahl E, Norheim OF, Klungsoyr K (2017). Increased risk of peripartum perinatal mortality in unplanned births outside an institution: A retrospective population-based study. Am J Obstet Gynecol.

[13] Grünebaum A, McCullough LB, Sapra KJ, Brent RL, Levene MI, Arabin B (2014). Early and total neonatal mortality in relation to birth setting in the United States, 2006-2009. Am J Obstet Gynecol.

[14] Vagle H, Haukeland GT, Dahl B, Aasheim V, Vik ES (2019). Emergency medical technicians' experiences with unplanned births outside institutions: A qualitative interview study. Nurs Open.

[15] NEMSIS Technical Assistance Center (2019). National EMS Database, NEMSIS Public Release Research Data Set V3.4.0, 2018 User Manual.

[16] Centers for Disease Control Prevention (2020). International Classification of Diseases, Tenth Revision, Clinical Modification (ICD-10-CM).

[17] SNOMED International. SNOMED CT United States Edition 2020 [Available from: https://www.nlm.nih.gov/healthit/snomedct/us_edition.html. Accessed 29 June 2020.

[18] Healthcare Cost and Utilization Project (HCUP) (2020). Clinical Classifications Software Refined (CCSR).

[19] National Highway Traffic Safety Administration (2005). National EMS Core Content.

[20] Cantwell R, Clutton-Brock T, Cooper G, Dawson A, Drife J, Garrod D (2011). Saving mothers' lives: Reviewing maternal deaths to make motherhood safer: 2006-2008. The Eighth Report of the Confidential Enquiries into Maternal Deaths in the United Kingdom. BJOG.

[21] Singh S, McGlennan A, England A, Simons R (2012). A validation study of the CEMACH recommended modified early obstetric warning system (MEOWS). Anaesthesia..

[22] Gunnarsson B, Fasting S, Skogvoll E, Smárason AK, Salvesen KÅ (2017). Why babies die in unplanned out-of-institution births: An enquiry into perinatal deaths in Norway 1999-2013. Acta Obstet Gynecol Scand.

[23] Robertson JF, Braude DA, Stonehocker J, Moreno J (2015). Prehospital breech delivery with fetal head entrapment - a case report and review. Prehosp Emerg Care.

[24] Wyckoff MH, Perlman JM (2004). Effective ventilation and temperature control are vital to outborn resuscitation. Prehosp Emerg Care.

[25] Loughney A, Collis R, Dastgir S (2006). Birth before arrival at delivery suite: Associations and consequences. Br J Midwifery.

[26] National Registry of Emergency Medical Technicians (2019). Recertification Guide.

[27] National Highway Traffic Safety Administration, Office of EMS (2009). National Emergency Medical Services Education Standards.

[28] Bateman DA, O'Bryan L, Nicholas SW, Heagarty MC (1994). Outcome of unattended out-of-hospital births in Harlem. Arch Pediatr Adolesc Med.

[29] Williams TA, Finn J, Celenza A, Teng T-H, Jacobs IG (2013). Paramedic identification of acute pulmonary edema in a metropolitan ambulance service. Prehosp Emerg Care.

[30] Christie A, Costa-Scorse B, Nicholls M, Jones P, Howie G (2016). Accuracy of working diagnosis by paramedics for patients presenting with dyspnoea. Emerg Med Australas.

[31] Coventry LL, Bremner AP, Williams TA, Jacobs IG, Finn J (2014). Symptoms of myocardial infarction: Concordance between paramedic and hospital records. Prehosp Emerg Care.

[32] Koivulahti O, Tommila M, Haavisto E (2020). The accuracy of preliminary diagnoses made by paramedics - a cross-sectional comparative study. Scand J Trauma Resusc Emerg Med.

[33] Martín-Rodríguez F, Sanz-García A, Medina-Lozano E, Castro Villamor M, Carbajosa Rodríguez V, Del Pozo VC, et al. The value of prehospital early warning scores to predict in-hospital clinical deterioration: A multicenter, observational base-ambulance study. Prehosp Emerg Care. 2021;25(5):597–606. 10.1080/10903127.2020.1813224 32820947

[34] Studnek JR, Browne LR, Shah MI, Fumo N, Hansen M, Lerner EB (2020). Validity of the pediatric early warning score and the bedside pediatric early warning score in classifying patients who require the resources of a higher level pediatric hospital. Prehosp Emerg Care.

