# Supplementary material for: Frequency and severity of prehospital obstetric events encountered by emergency medical services in the United States
Source: BMC Pregnancy Childbirth. 2021 Sep 24;21:655. doi: 10.1186/s12884-021-04129-1 (PMC8464145; doi:10.1186/s12884-021-04129-1)
Supplement: Supplementary file 1 — Additional file 1: Table S1. Identification of EMS 911 activations for a potential obstetric event in patients of childbearing age (12-50 years). [file 12884_2021_4129_MOESM1_ESM.docx]

**Table S1.** Identification of EMS 911 activations for a potential obstetric event in patients of childbearing age (12-50 years).

| **Criteria** | **Variable name** | **Included values** | **Records found*** |
| --- | --- | --- | --- |
| Dispatch reason | eDispatch.01 | 2301057 (Pregnancy/childbirth/miscarriage) | 84,549 |
| Primary symptom | eSituation.09 | ICD-10-CM codes beginning with: O, P, Z32, Z33, Z34, Z36, Z37, Z38, Z39, Z3A | 24,513 |
| Associated symptoms | eSituation.10 (multi-select) | ICD-10-CM codes beginning with: O, P, Z32, Z33, Z34, Z36, Z37, Z38, Z39, Z3A | 2,174 |
| Primary impression | eSituation.11 | ICD-10-CM codes beginning with: O, P, Z32, Z33, Z34, Z36, Z37, Z38, Z39, Z3A | 58,083 |
| Secondary impressions | eSituation.12  (multi-select) | ICD-10-CM codes beginning with: O, P, Z32, Z33, Z34, Z36, Z37, Z38, Z39, Z3A | 11,953 |
| Procedures | eProcedures.03  (multi-select) | 236996005 (Abdominal uterine fundal massage)  42550007 (Catheterization of umbilical vein)  409006000 (Delivery care)  409012005 (Assess delivery care)  56620000 (Delivery of placenta following delivery of infant outside of hospital)  236973005 (Delivery procedure/obstetric delivery)  85403009 (Delivery, medical personnel present)  424432007 (Epidural catheter maintenance)  36708009 (External fetal monitor surveillance)  408987002 (Newborn care assessment)  408989004 (Newborn care management)  423589000 (Newborn continuous physical assessment)  177184002 (Normal delivery procedure)  8390008 (Routine care of newborn)  313006001 (Ultrasonic doppler for fetal heart sounds)  233519002 (Umbilical artery cannula insertion)  238247000 (Umbilical cord procedure)  274504000 (Umbilical vessel catheterization)  700000006 (Vaginal delivery of fetus)  22633006 (Vaginal delivery, medical personnel present)  16310003 (Ultrasound)  18540005 (Suction of newborn)  236994008 (Placental delivery procedure)  238248005 (Umbilical cord clamping)  408806008 (Fetal heart monitoring using handheld doppler)  447214008 (Ligation of umbilical cord of fetus) | 1,610 |
| Protocols used | eProcotols.01  (multi-select) | 9914155 (OB/GYN-Childbirth/Labor/Delivery)  9914157 (OB/GYN-Eclampsia)  9914161 (OB/GYN-Pregnancy Related Emergencies)  9914163 (OB/GYN-Post-partum Hemorrhage)  9914133 (Medical-Newborn/Neonatal Resuscitation)  *Note: Events with only 9914159 (OB/GYN-Gynecologic Emergencies) were excluded* | 16,238 |

Abbreviations: EMS, emergency medical services; ICD-10-CM, International Classification of Diseases, Tenth Revision, Clinical Modification; OB/GYN, obstetrics/gynecology.

*Records may have been identified by multiple criteria and are not mutually exclusive or additive. There was a grand total of 107,771 unique records identified.
