# Supplementary material for: Frequency and severity of prehospital obstetric events encountered by emergency medical services in the United States
Source: BMC Pregnancy Childbirth. 2021 Sep 24;21:655. doi: 10.1186/s12884-021-04129-1 (PMC8464145; doi:10.1186/s12884-021-04129-1)
Supplement: Supplementary file 2 — Additional file 2: Table S2. Definition of the 14 pre-specified obstetric conditions and events. [file 12884_2021_4129_MOESM2_ESM.docx]

**Table S2.** Definition of the 14 pre-specified obstetric conditions and events.

| **Item** | **NEMSIS Variable** | **Included values*** |
| --- | --- | --- |
| Ectopic or molar pregnancy and complications | eSituation.09  eSituation.10  eSituation.11  eSituation.12 | O000 Abdominal pregnancy  O0000 Abdominal pregnancy without intrauterine pregnancy  O0001 Abdominal pregnancy with intrauterine pregnancy  O001 Tubal pregnancy  O0010 Tubal pregnancy without intrauterine pregnancy  O00101 Right tubal pregnancy without intrauterine pregnancy  O00102 Left tubal pregnancy without intrauterine pregnancy  O00109 Unspecified tubal pregnancy without intrauterine pregnancy  O0011 Tubal pregnancy with intrauterine pregnancy  O00111 Right tubal pregnancy with intrauterine pregnancy  O00112 Left tubal pregnancy with intrauterine pregnancy  O00119 Unspecified tubal pregnancy with intrauterine pregnancy  O002 Ovarian pregnancy  O0020 Ovarian pregnancy without intrauterine pregnancy  O00201 Right ovarian pregnancy without intrauterine pregnancy  O00202 Left ovarian pregnancy without intrauterine pregnancy  O00209 Unspecified ovarian pregnancy without intrauterine pregnancy  O0021 Ovarian pregnancy with intrauterine pregnancy  O00211 Right ovarian pregnancy with intrauterine pregnancy  O00212 Left ovarian pregnancy without intrauterine pregnancy  O00219 Unspecified ovarian pregnancy with intrauterine pregnancy  O008 Other ectopic pregnancy  O0080 Other ectopic pregnancy without intrauterine pregnancy  O0081 Other ectopic pregnancy with intrauterine pregnancy  O009 Ectopic pregnancy unspecified  O0090 Unspecified ectopic pregnancy without intrauterine pregnancy  O0091 Unspecified ectopic pregnancy with intrauterine pregnancy  O3670X0-9 Maternal care for viable fetus in abdominal pregnancy, unspecified trimester  O3671X0-9 Maternal care for viable fetus in abdominal pregnancy, first trimester  O3672X0-9 Maternal care for viable fetus in abdominal pregnancy, second trimester  O3673X0-9 Maternal care for viable fetus in abdominal pregnancy, third trimester  O010 Classical hydatidiform mole  O011 Incomplete and partial hydatidiform mole  O019 Hydatidiform mole, unspecified  O020 Blighted ovum and nonhydatidiform mole  O021 Missed abortion  O0281 Inapprop chg quantitav hCG in early pregnancy  O0289 Other abnormal products of conception  O029 Abnormal product of conception, unspecified  O0881 Cardiac arrest following an ectopic and molar pregnancy  O0882 Sepsis following ectopic and molar pregnancy  O080 Genitl trct and pelvic infct fol ectopic and molar pregnancy  O081 Delayed or excess hemor fol ectopic and molar pregnancy  O082 Embolism following ectopic and molar pregnancy  O083 Shock following ectopic and molar pregnancy  O084 Renal failure following ectopic and molar pregnancy  O085 Metabolic disorders following an ectopic and molar pregnancy  O086 Damage to pelvic organs and tiss fol an ect and molar preg  O087 Oth venous comp following an ectopic and molar pregnancy  O0883 Urinary tract infection fol an ectopic and molar pregnancy  O0889 Other complications following an ectopic and molar pregnancy  O089 Unsp complication following an ectopic and molar pregnancy |
| Spontaneous or induced abortion and complications | eSituation.09  eSituation.10  eSituation.11  eSituation.12 | O0336 Cardiac arrest following incomplete spontaneous abortion  O0386 Cardiac arrest following complete or unspecified spontaneous abortion  O0337 Sepsis following incomplete spontaneous abortion  O0387 Sepsis following complete or unspecified spontaneous abortion  O030 Genitl trct and pelvic infection fol incmpl spon abortion  O031 Delayed or excessive hemor following incmpl spon abortion  O032 Embolism following incomplete spontaneous abortion  O0330 Unsp complication following incomplete spontaneous abortion  O0331 Shock following incomplete spontaneous abortion  O0332 Renal failure following incomplete spontaneous abortion  O0333 Metabolic disorder following incomplete spontaneous abortion  O0334 Damage to pelvic organs following incomplete spon abortion  O0335 Oth venous comp following incomplete spontaneous abortion  O0338 Urinary tract infection following incomplete spon abortion  O0339 Incomplete spontaneous abortion with other complications  O034 Incomplete spontaneous abortion without complication  O035 Genitl trct and pelvic infct fol complete or unsp spon abort  O036 Delayed or excess hemor fol complete or unsp spon abortion  O037 Embolism following complete or unsp spontaneous abortion  O0380 Unsp comp following complete or unsp spontaneous abortion  O0381 Shock following complete or unspecified spontaneous abortion  O0382 Renal failure following complete or unsp spon abortion  O0383 Metabolic disorder following complete or unsp spon abortion  O0384 Damage to pelvic organs fol complete or unsp spon abortion  O0385 Oth venous comp following complete or unsp spon abortion  O0388 Urinary tract infection fol complete or unsp spon abortion  O0389 Complete or unsp spontaneous abortion with oth complications  O039 Complete or unsp spontaneous abortion without complication  O0486 Cardiac arrest following (induced) termination of pregnancy  O0736 Cardiac arrest following failed attempted termination of pregnancy  O0487 Sepsis following (induced) termination of pregnancy  O0737 Sepsis following failed attempted termination of pregnancy  O045 Genitl trct and pelvic infct fol (induced) term of pregnancy  O046 Delayed or excess hemor fol (induced) term of pregnancy  O047 Embolism following (induced) termination of pregnancy  O0480 (Induced) termination of pregnancy with unsp complications  O0481 Shock following (induced) termination of pregnancy  O0482 Renal failure following (induced) termination of pregnancy  O0483 Metabolic disorder following (induced) term of pregnancy  O0484 Damage to pelvic organs fol (induced) term of pregnancy  O0485 Oth venous comp following (induced) termination of pregnancy  O0488 Urinary tract infection fol (induced) term of pregnancy  O0489 (Induced) termination of pregnancy with other complications  O070 Genitl trct and pelvic infct fol failed attempt term of preg  O071 Delayed or excess hemor fol failed attempt term of pregnancy  O072 Embolism following failed attempted termination of pregnancy  O0730 Failed attempted termination of pregnancy w unsp comp  O0731 Shock following failed attempted termination of pregnancy  O0732 Renal failure following failed attempted term of pregnancy  O0733 Metabolic disorder fol failed attempt term of pregnancy  O0734 Damage to pelvic organs fol failed attempt term of pregnancy  O0735 Oth venous comp following failed attempted term of pregnancy  O0738 Urinary tract infection fol failed attempt term of pregnancy  O0739 Failed attempted termination of pregnancy w oth comp  O074 Failed attempted termination of pregnancy w/o complication  Z332 Encounter for elective termination of pregnancy |
| Early, first or unspecified trimester hemorrhage | eSituation.09  eSituation.10  eSituation.11  eSituation.12 | O200 Threatened abortion  O208 Other hemorrhage in early pregnancy  O209 Hemorrhage in early pregnancy, unspecified  O26851 Spotting complicating pregnancy, first trimester  O26859 Spotting complicating pregnancy, unspecified trimester  O46001 Antepartum hemorrhage w coag defect, unsp, first trimester  O46009 Antepartum hemorrhage w coag defect, unsp, unsp trimester  O46011 Antepartum hemorrhage with afibrinogenemia, first trimester  O46019 Antepartum hemorrhage with afibrinogenemia, unsp trimester  O46021 Antepart hemorrhage w dissem intravasc coag, first trimester  O46029 Antepart hemorrhage w dissem intravasc coag, unsp trimester  O46091 Antepartum hemorrhage w oth coag defect, first trimester  O46099 Antepartum hemorrhage w oth coag defect, unsp trimester  O468X1 Other antepartum hemorrhage, first trimester  O468X9 Other antepartum hemorrhage, unspecified trimester  O4690 Antepartum hemorrhage, unspecified, unspecified trimester  O4691 Antepartum hemorrhage, unspecified, first trimester  O26852 Spotting complicating pregnancy, second trimester  O26853 Spotting complicating pregnancy, third trimester  O46002 Antepartum hemorrhage w coag defect, unsp, second trimester  O46003 Antepartum hemorrhage w coag defect, unsp, third trimester  O46012 Antepartum hemorrhage with afibrinogenemia, second trimester  O46013 Antepartum hemorrhage with afibrinogenemia, third trimester  O46022 Antepart hemor w dissem intravasc coag, second trimester  O46023 Antepart hemorrhage w dissem intravasc coag, third trimester  O46092 Antepartum hemorrhage w oth coag defect, second trimester  O46093 Antepartum hemorrhage w oth coag defect, third trimester  O468X2 Other antepartum hemorrhage, second trimester  O468X3 Other antepartum hemorrhage, third trimester  O4692 Antepartum hemorrhage, unspecified, second trimester  O4693 Antepartum hemorrhage, unspecified, third trimester  O4410 Complete placenta previa with hemorrhage, unspecified trimester  O4411 Complete placenta previa with hemorrhage, first trimester  O4430 Partial placenta previa with hemorrhage, unspecified trimester  O4431 Partial placenta previa with hemorrhage, first trimester  O4450 Low lying placenta with hemorrhage, unspecified trimester  O4451 Low lying placenta with hemorrhage, first trimester  O45001 Premature separation of placenta with coagulation defect, unspecified, first trimester  O45009 Premature separation of placenta with coagulation defect, unspecified, unspecified trimester  O45011 Premature separation of placenta with afibrinogenemia, first trimester  O45019 Premature separation of placenta with afibrinogenemia, unspecified trimester  O45021 Premature separation of placenta with disseminated intravascular coagulation, first trimester  O45029 Premature separation of placenta with disseminated intravascular coagulation, unspecified trimester  O45091 Premature separation of placenta with other coagulation defect, first trimester  O45099 Premature separation of placenta with other coagulation defect, unspecified trimester  O458X1 Other premature separation of placenta, first trimester  O458X9 Other premature separation of placenta, unspecified trimester  O4590 Premature separation of placenta, unspecified, unspecified trimester  O4591 Premature separation of placenta, unspecified, first trimester  O4412 Complete placenta previa with hemorrhage, second trimester  O4413 Complete placenta previa with hemorrhage, third trimester  O4432 Partial placenta previa with hemorrhage, second trimester  O4433 Partial placenta previa with hemorrhage, third trimester  O4452 Low lying placenta with hemorrhage, second trimester  O4453 Low lying placenta with hemorrhage, third trimester  O45002 Premature separation of placenta with coagulation defect, unspecified, second trimester  O45003 Premature separation of placenta with coagulation defect, unspecified, third trimester  O45012 Premature separation of placenta with afibrinogenemia, second trimester  O45013 Premature separation of placenta with afibrinogenemia, third trimester  O45022 Premature separation of placenta with disseminated intravascular coagulation, second trimester  O45023 Premature separation of placenta with disseminated intravascular coagulation, third trimester  O45092 Premature separation of placenta with other coagulation defect, second trimester  O45093 Premature separation of placenta with other coagulation defect, third trimester  O458X2 Other premature separation of placenta, second trimester  O458X3 Other premature separation of placenta, third trimester  O4592 Premature separation of placenta, unspecified, second trimester  O4593 Premature separation of placenta, unspecified, third trimester |
| Eclampsia, preeclampsia, and hypertensive conditions | eSituation.09  eSituation.10  eSituation.11  eSituation.12 | O10011 Pre-existing essential hypertension complicating pregnancy, first trimester  O10012 Pre-existing essential hypertension complicating pregnancy, second trimester  O10013 Pre-existing essential hypertension complicating pregnancy, third trimester  O10019 Pre-existing essential hypertension complicating pregnancy, unspecified trimester  O1002 Pre-existing essential hypertension complicating childbirth  O1003 Pre-existing essential hypertension complicating the puerperium  O10911 Unspecified pre-existing hypertension complicating pregnancy, first trimester  O10912 Unspecified pre-existing hypertension complicating pregnancy, second trimester  O10913 Unspecified pre-existing hypertension complicating pregnancy, third trimester  O10919 Unspecified pre-existing hypertension complicating pregnancy, unspecified trimester  O1092 Unspecified pre-existing hypertension complicating childbirth  O1093 Unspecified pre-existing hypertension complicating the puerperium  O161 Unspecified maternal hypertension, first trimester  O162 Unspecified maternal hypertension, second trimester  O163 Unspecified maternal hypertension, third trimester  O164 Unspecified maternal hypertension, complicating childbirth  O165 Unspecified maternal hypertension, complicating the puerperium  O169 Unspecified maternal hypertension, unspecified trimester  O10111 Pre-existing hypertensive heart disease complicating pregnancy, first trimester  O10112 Pre-existing hypertensive heart disease complicating pregnancy, second trimester  O10113 Pre-existing hypertensive heart disease complicating pregnancy, third trimester  O10119 Pre-existing hypertensive heart disease complicating pregnancy, unspecified trimester  O1012 Pre-existing hypertensive heart disease complicating childbirth  O1013 Pre-existing hypertensive heart disease complicating the puerperium  O10211 Pre-existing hypertensive chronic kidney disease complicating pregnancy, first trimester  O10212 Pre-existing hypertensive chronic kidney disease complicating pregnancy, second trimester  O10213 Pre-existing hypertensive chronic kidney disease complicating pregnancy, third trimester  O10219 Pre-existing hypertensive chronic kidney disease complicating pregnancy, unspecified trimester  O1022 Pre-existing hypertensive chronic kidney disease complicating childbirth  O1023 Pre-existing hypertensive chronic kidney disease complicating the puerperium  O10311 Pre-existing hypertensive heart and chronic kidney disease complicating pregnancy, first trimester  O10312 Pre-existing hypertensive heart and chronic kidney disease complicating pregnancy, second trimester  O10313 Pre-existing hypertensive heart and chronic kidney disease complicating pregnancy, third trimester  O10319 Pre-existing hypertensive heart and chronic kidney disease complicating pregnancy, unspecified trimester  O1032 Pre-existing hypertensive heart and chronic kidney disease complicating childbirth  O1033 Pre-existing hypertensive heart and chronic kidney disease complicating the puerperium  O10411 Pre-existing secondary hypertension complicating pregnancy, first trimester  O10412 Pre-existing secondary hypertension complicating pregnancy, second trimester  O10413 Pre-existing secondary hypertension complicating pregnancy, third trimester  O10419 Pre-existing secondary hypertension complicating pregnancy, unspecified trimester  O1042 Pre-existing secondary hypertension complicating childbirth  O1043 Pre-existing secondary hypertension complicating the puerperium  O111 Pre-existing hypertension with pre-eclampsia, first trimester  O112 Pre-existing hypertension with pre-eclampsia, second trimester  O113 Pre-existing hypertension with pre-eclampsia, third trimester  O114 Pre-existing hypertension with pre-eclampsia, complicating childbirth  O115 Pre-existing hypertension with pre-eclampsia, complicating the puerperium  O119 Pre-existing hypertension with pre-eclampsia, unspecified trimester  O131 Gestational [pregnancy-induced] hypertension without significant proteinuria, first trimester  O132 Gestational [pregnancy-induced] hypertension without significant proteinuria, second trimester  O133 Gestational [pregnancy-induced] hypertension without significant proteinuria, third trimester  O134 Gestational [pregnancy-induced] hypertension without significant proteinuria, complicating childbirth  O135 Gestational [pregnancy-induced] hypertension without significant proteinuria, complicating the puerperium  O139 Gestational [pregnancy-induced] hypertension without significant proteinuria, unspecified trimester  O1400 Mild to moderate pre-eclampsia, unspecified trimester  O1402 Mild to moderate pre-eclampsia, second trimester  O1403 Mild to moderate pre-eclampsia, third trimester  O1404 Mild to moderate pre-eclampsia, complicating childbirth  O1405 Mild to moderate pre-eclampsia, complicating the puerperium  O1410 Severe pre-eclampsia, unspecified trimester  O1412 Severe pre-eclampsia, second trimester  O1413 Severe pre-eclampsia, third trimester  O1414 Severe pre-eclampsia complicating childbirth  O1415 Severe pre-eclampsia, complicating the puerperium  O1420 HELLP syndrome (HELLP), unspecified trimester  O1422 HELLP syndrome (HELLP), second trimester  O1423 HELLP syndrome (HELLP), third trimester  O1424 HELLP syndrome, complicating childbirth  O1425 HELLP syndrome, complicating the puerperium  O1490 Unspecified pre-eclampsia, unspecified trimester  O1492 Unspecified pre-eclampsia, second trimester  O1493 Unspecified pre-eclampsia, third trimester  O1494 Unspecified pre-eclampsia, complicating childbirth  O1495 Unspecified pre-eclampsia, complicating the puerperium  O1500 Eclampsia complicating pregnancy, unspecified trimester  O1502 Eclampsia complicating pregnancy, second trimester  O1503 Eclampsia complicating pregnancy, third trimester  O151 Eclampsia complicating childbirth  O152 Eclampsia complicating the puerperium  O159 Eclampsia, unspecified as to time period |
| Early or threatened labor | eSituation.09  eSituation.10  eSituation.11  eSituation.12 | O26872 Cervical shortening, second trimester  O26873 Cervical shortening, third trimester  O26879 Cervical shortening, unspecified trimester  O3430 Maternal care for cervical incompetence, unsp trimester  O3431 Maternal care for cervical incompetence, first trimester  O3432 Maternal care for cervical incompetence, second trimester  O3433 Maternal care for cervical incompetence, third trimester  O4200 Prem ROM, onset labor w/n 24 hr of rupt, unsp weeks of gest  O42011 Pretrm prem ROM, onset labor w/n 24 hours of rupt, first tri  O42012 Pretrm prem ROM, onset labor w/n 24 hours of rupt, 2nd tri  O42013 Pretrm prem ROM, onset labor w/n 24 hours of rupt, third tri  O42019 Pretrm prem ROM, onset labor w/n 24 hours of rupt, unsp tri  O4210 Prem ROM, onset labor > 24 hr fol rupt, unsp weeks of gest  O42111 Pretrm prem ROM, onset labor > 24 hours fol rupt, first tri  O42112 Pretrm prem ROM, onset labor > 24 hours fol rupt, second tri  O42113 Pretrm prem ROM, onset labor > 24 hours fol rupt, third tri  O42119 Pretrm prem ROM, onset labor > 24 hours fol rupt, unsp tri  O4290 Prem ROM, 7th0 betw rupt & onst labr, unsp weeks of gest  O42911 Pretrm prem ROM, unsp time betw rupt and onset labr, 1st tri  O42912 Pretrm prem ROM, unsp time betw rupt and onset labr, 2nd tri  O42913 Pretrm prem ROM, unsp time betw rupt and onst labr, 3rd tri  O42919 Pretrm prem ROM, unsp time betw rupt and onst labr, unsp tri  O4700 False labor before 37 completed weeks of gest, unsp tri  O4702 False labor before 37 completed weeks of gest, second tri  O4703 False labor before 37 completed weeks of gest, third tri  O471 False labor at or after 37 completed weeks of gestation  O479 False labor, unspecified  O6000 Preterm labor without delivery, unspecified trimester  O6002 Preterm labor without delivery, second trimester  O6003 Preterm labor without delivery, third trimester  O6010X0-9 Preterm labor w preterm delivery, unsp trimester  O6012X0-9 Preterm labor second tri w preterm delivery second tri  O6013X0-9 Preterm labor second tri w preterm delivery third tri  O6014X0-9 Preterm labor third tri w preterm delivery third tri  O6020X0-9 Term delivery with preterm labor, unspecified trimester  O6022X0-9 Term delivery with preterm labor, second trimester  O6023X0-9 Term delivery with preterm labor, third trimester |
| Malposition, disproportion, or other labor complications | eSituation.09  eSituation.10  eSituation.11  eSituation.12 | O771 Fetal stress in labor or delivery due to drug administration  O320XX0-9 Maternal care for unstable lie  O321XX0-9 Maternal care for breech presentation  O322XX0-9 Maternal care for transverse and oblique lie  O323XX0-9 Maternal care for face, brow and chin presentation  O324XX0-9 Maternal care for high head at term  O326XX0-9 Maternal care for compound presentation  O328XX0-9 Maternal care for oth malpresentation of fetus  O329XX0-9 Maternal care for malpresentation of fetus, unsp  O330 Matern care for disproprtn d/t deformity of matern pelv bone  O331 Matern care for disproprtn d/t generally contracted pelvis  O332 Maternal care for disproprtn due to inlet contrctn of pelvis  O333XX0-9 Matern care for disproprtn d/t outlet contrctn of pelv  O334XX0-9 Matern care for disproprtn of mix matern & fetl origin  O335XX0-9 Matern care for disproprtn d/t unusually large fetus  O336XX0-9 Matern care for disproprtn due to hydrocephalic fetus  O337 Maternal care for disproportion due to other fetal deformiti  O337XX0-9 Maternal care for disproprtn due to other fetal deform  O338 Maternal care for disproportion of other origin  O339 Maternal care for disproportion, unspecified  O610 Failed medical induction of labor  O611 Failed instrumental induction of labor  O618 Other failed induction of labor  O619 Failed induction of labor, unspecified  O620 Primary inadequate contractions  O621 Secondary uterine inertia  O622 Other uterine inertia  O623 Precipitate labor  O624 Hypertonic, incoordinate, and prolonged uterine contractions  O628 Other abnormalities of forces of labor  O629 Abnormality of forces of labor, unspecified  O630 Prolonged first stage (of labor)  O631 Prolonged second stage (of labor)  O632 Delayed delivery of second twin, triplet, etc.  O639 Long labor, unspecified  O640XX0-9 Obstructed labor due to incmpl rotation of fetal head  O641XX0-9 Obstructed labor due to breech presentation  O642XX0-9 Obstructed labor due to face presentation  O643XX0-9 Obstructed labor due to brow presentation  O644XX0-9 Obstructed labor due to shoulder presentation  O645XX0-9 Obstructed labor due to compound presentation  O648XX0-9 Obstructed labor due to oth malposition and malpresent,  O649XX0-9 Obstructed labor due to malpos and malpresent  O650 Obstructed labor due to deformed pelvis  O651 Obstructed labor due to generally contracted pelvis  O652 Obstructed labor due to pelvic inlet contraction  O653 Obst labor due to pelvic outlet and mid-cavity contrctn  O654 Obstructed labor due to fetopelvic disproportion, unsp  O655 Obstructed labor due to abnlt of maternal pelvic organs  O658 Obstructed labor due to other maternal pelvic abnormalities  O659 Obstructed labor due to maternal pelvic abnormality, unsp  O660 Obstructed labor due to shoulder dystocia  O661 Obstructed labor due to locked twins  O662 Obstructed labor due to unusually large fetus  O663 Obstructed labor due to other abnormalities of fetus  O6640 Failed trial of labor, unspecified  O6641 Failed attempt vaginal birth after previous cesarean del  O665 Attempted application of vacuum extractor and forceps  O666 Obstructed labor due to other multiple fetuses  O668 Other specified obstructed labor  O669 Obstructed labor, unspecified  O68 Labor and delivery comp by abnlt of fetal acid-base balance  O690XX0-9 Labor and delivery complicated by prolapse of cord  O691XX0-9 Labor and delivery comp by cord around neck, w comprsn  O692XX0-9 Labor and del comp by oth cord entangle, w comprsn  O693XX0-9 Labor and delivery complicated by short cord  O694XX0-9 Labor and delivery complicated by vasa previa  O695XX0-9 Labor and delivery comp by vascular lesion of cord  O6981X0-9 Labor and del comp by cord around neck, w/o comprsn  O6982X0-9 Labor and del comp by oth cord entangle, w/o comprsn  O6989X0-9 Labor and delivery complicated by oth cord comp  O699XX0-9 Labor and delivery complicated by cord comp,  O778 Labor and delivery comp by oth evidence of fetal stress  O779 Labor and delivery complicated by fetal stress, unspecified |
| Prolapsed cord | eSituation.09  eSituation.10  eSituation.11  eSituation.12 | O690XX0 Labor and delivery complicated by prolapse of cord, unsp  O690XX1 Labor and delivery complicated by prolapse of cord, fetus 1  O690XX2 Labor and delivery complicated by prolapse of cord, fetus 2  O690XX3 Labor and delivery complicated by prolapse of cord, fetus 3  O690XX4 Labor and delivery complicated by prolapse of cord, fetus 4  O690XX5 Labor and delivery complicated by prolapse of cord, fetus 5  O690XX9 Labor and delivery complicated by prolapse of cord, oth |
| Nuchal cord | eSituation.09  eSituation.10  eSituation.11  eSituation.12 | O691XX0 Labor and delivery comp by cord around neck, w comprsn, unsp  O691XX1 Labor and del comp by cord around neck, w comprsn, fetus 1  O691XX2 Labor and del comp by cord around neck, w comprsn, fetus 2  O691XX3 Labor and del comp by cord around neck, w comprsn, fetus 3  O691XX4 Labor and del comp by cord around neck, w comprsn, fetus 4  O691XX5 Labor and del comp by cord around neck, w comprsn, fetus 5  O691XX9 Labor and delivery comp by cord around neck, w comprsn, oth  O6981X0 Labor and del comp by cord around neck, w/o comprsn, unsp  O6981X1 Labor and del comp by cord around neck, w/o comprsn, fetus 1  O6981X2 Labor and del comp by cord around neck, w/o comprsn, fetus 2  O6981X3 Labor and del comp by cord around neck, w/o comprsn, fetus 3  O6981X4 Labor and del comp by cord around neck, w/o comprsn, fetus 4  O6981X5 Labor and del comp by cord around neck, w/o comprsn, fetus 5  O6981X9 Labor and del comp by cord around neck, w/o comprsn, oth |
| Non-cephalic presentation | eSituation.09  eSituation.10  eSituation.11  eSituation.12 | O320XX0 Maternal care for unstable lie, not applicable or unsp  O320XX1 Maternal care for unstable lie, fetus 1  O320XX2 Maternal care for unstable lie, fetus 2  O320XX3 Maternal care for unstable lie, fetus 3  O320XX4 Maternal care for unstable lie, fetus 4  O320XX5 Maternal care for unstable lie, fetus 5  O320XX9 Maternal care for unstable lie, other fetus  O321XX0 Maternal care for breech presentation, unsp  O321XX1 Maternal care for breech presentation, fetus 1  O321XX2 Maternal care for breech presentation, fetus 2  O321XX3 Maternal care for breech presentation, fetus 3  O321XX4 Maternal care for breech presentation, fetus 4  O321XX5 Maternal care for breech presentation, fetus 5  O321XX9 Maternal care for breech presentation, other fetus  O322XX0 Maternal care for transverse and oblique lie, unsp  O322XX1 Maternal care for transverse and oblique lie, fetus 1  O322XX2 Maternal care for transverse and oblique lie, fetus 2  O322XX3 Maternal care for transverse and oblique lie, fetus 3  O322XX4 Maternal care for transverse and oblique lie, fetus 4  O322XX5 Maternal care for transverse and oblique lie, fetus 5  O322XX9 Maternal care for transverse and oblique lie, other fetus  O323XX0 Maternal care for face, brow and chin presentation, unsp  O323XX1 Maternal care for face, brow and chin presentation, fetus 1  O323XX2 Maternal care for face, brow and chin presentation, fetus 2  O323XX3 Maternal care for face, brow and chin presentation, fetus 3  O323XX4 Maternal care for face, brow and chin presentation, fetus 4  O323XX5 Maternal care for face, brow and chin presentation, fetus 5  O323XX9 Maternal care for face, brow and chin presentation, oth  O324XX0 Maternal care for high head at term, not applicable or unsp  O324XX1 Maternal care for high head at term, fetus 1  O324XX2 Maternal care for high head at term, fetus 2  O324XX3 Maternal care for high head at term, fetus 3  O324XX4 Maternal care for high head at term, fetus 4  O324XX5 Maternal care for high head at term, fetus 5  O324XX9 Maternal care for high head at term, other fetus  O326XX0 Maternal care for compound presentation, unsp  O326XX1 Maternal care for compound presentation, fetus 1  O326XX2 Maternal care for compound presentation, fetus 2  O326XX3 Maternal care for compound presentation, fetus 3  O326XX4 Maternal care for compound presentation, fetus 4  O326XX5 Maternal care for compound presentation, fetus 5  O326XX9 Maternal care for compound presentation, other fetus  O328XX0 Maternal care for oth malpresentation of fetus, unsp  O328XX1 Maternal care for other malpresentation of fetus, fetus 1  O328XX2 Maternal care for other malpresentation of fetus, fetus 2  O328XX3 Maternal care for other malpresentation of fetus, fetus 3  O328XX4 Maternal care for other malpresentation of fetus, fetus 4  O328XX5 Maternal care for other malpresentation of fetus, fetus 5  O328XX9 Maternal care for oth malpresentation of fetus, other fetus  O329XX0 Maternal care for malpresentation of fetus, unsp, unsp  O329XX1 Maternal care for malpresentation of fetus, unsp, fetus 1  O329XX2 Maternal care for malpresentation of fetus, unsp, fetus 2  O329XX3 Maternal care for malpresentation of fetus, unsp, fetus 3  O329XX4 Maternal care for malpresentation of fetus, unsp, fetus 4  O329XX5 Maternal care for malpresentation of fetus, unsp, fetus 5  O329XX9 Maternal care for malpresentation of fetus, unsp, oth fetus  O640XX0 Obstructed labor due to incmpl rotation of fetal head, unsp  O640XX1 Obst labor due to incmpl rotation of fetal head, fetus 1  O640XX2 Obst labor due to incmpl rotation of fetal head, fetus 2  O640XX3 Obst labor due to incmpl rotation of fetal head, fetus 3  O640XX4 Obst labor due to incmpl rotation of fetal head, fetus 4  O640XX5 Obst labor due to incmpl rotation of fetal head, fetus 5  O640XX9 Obstructed labor due to incmpl rotation of fetal head, oth  O641XX0 Obstructed labor due to breech presentation, unsp  O641XX1 Obstructed labor due to breech presentation, fetus 1  O641XX2 Obstructed labor due to breech presentation, fetus 2  O641XX3 Obstructed labor due to breech presentation, fetus 3  O641XX4 Obstructed labor due to breech presentation, fetus 4  O641XX5 Obstructed labor due to breech presentation, fetus 5  O641XX9 Obstructed labor due to breech presentation, other fetus  O642XX0 Obstructed labor due to face presentation, unsp  O642XX1 Obstructed labor due to face presentation, fetus 1  O642XX2 Obstructed labor due to face presentation, fetus 2  O642XX3 Obstructed labor due to face presentation, fetus 3  O642XX4 Obstructed labor due to face presentation, fetus 4  O642XX5 Obstructed labor due to face presentation, fetus 5  O642XX9 Obstructed labor due to face presentation, other fetus  O643XX0 Obstructed labor due to brow presentation, unsp  O643XX1 Obstructed labor due to brow presentation, fetus 1  O643XX2 Obstructed labor due to brow presentation, fetus 2  O643XX3 Obstructed labor due to brow presentation, fetus 3  O643XX4 Obstructed labor due to brow presentation, fetus 4  O643XX5 Obstructed labor due to brow presentation, fetus 5  O643XX9 Obstructed labor due to brow presentation, other fetus  O644XX0 Obstructed labor due to shoulder presentation, unsp  O644XX1 Obstructed labor due to shoulder presentation, fetus 1  O644XX2 Obstructed labor due to shoulder presentation, fetus 2  O644XX3 Obstructed labor due to shoulder presentation, fetus 3  O644XX4 Obstructed labor due to shoulder presentation, fetus 4  O644XX5 Obstructed labor due to shoulder presentation, fetus 5  O644XX9 Obstructed labor due to shoulder presentation, other fetus  O645XX0 Obstructed labor due to compound presentation, unsp  O645XX1 Obstructed labor due to compound presentation, fetus 1  O645XX2 Obstructed labor due to compound presentation, fetus 2  O645XX3 Obstructed labor due to compound presentation, fetus 3  O645XX4 Obstructed labor due to compound presentation, fetus 4  O645XX5 Obstructed labor due to compound presentation, fetus 5  O645XX9 Obstructed labor due to compound presentation, other fetus  O648XX0 Obstructed labor due to oth malposition and malpresent, unsp  O648XX1 Obstructed labor due to oth malpos and malpresent, fetus 1  O648XX2 Obstructed labor due to oth malpos and malpresent, fetus 2  O648XX3 Obstructed labor due to oth malpos and malpresent, fetus 3  O648XX4 Obstructed labor due to oth malpos and malpresent, fetus 4  O648XX5 Obstructed labor due to oth malpos and malpresent, fetus 5  O648XX9 Obstructed labor due to oth malposition and malpresent, oth  O649XX0 Obstructed labor due to malpos and malpresent, unsp, unsp  O649XX1 Obstructed labor due to malpos and malpresent, unsp, fetus 1  O649XX2 Obstructed labor due to malpos and malpresent, unsp, fetus 2  O649XX3 Obstructed labor due to malpos and malpresent, unsp, fetus 3  O649XX4 Obstructed labor due to malpos and malpresent, unsp, fetus 4  O649XX5 Obstructed labor due to malpos and malpresent, unsp, fetus 5  O649XX9 Obstructed labor due to malpos and malpresent, unsp, oth |
| Intra- and post-partum hemorrhage | eSituation.09  eSituation.10  eSituation.11  eSituation.12 | O670 Intrapartum hemorrhage with coagulation defect  O678 Other intrapartum hemorrhage  O679 Intrapartum hemorrhage, unspecified  O720 Third-stage hemorrhage  O721 Other immediate postpartum hemorrhage  O722 Delayed and secondary postpartum hemorrhage |
| Out-of-hospital birth/delivery | eSituation.09  eSituation.10  eSituation.11  eSituation.12 | O80 Encounter for full-term uncomplicated delivery  Z390 Encntr for care and exam of mother immediately after del  Z370 Single live birth  Z371 Single stillbirth  Z372 Twins, both liveborn  Z373 Twins, one liveborn and one stillborn  Z374 Twins, both stillborn  Z3750 Multiple births, unspecified, all liveborn  Z3751 Triplets, all liveborn  Z3752 Quadruplets, all liveborn  Z3753 Quintuplets, all liveborn  Z3754 Sextuplets, all liveborn  Z3759 Other multiple births, all liveborn  Z3760 Multiple births, unspecified, some liveborn  Z3761 Triplets, some liveborn  Z3762 Quadruplets, some liveborn  Z3763 Quintuplets, some liveborn  Z3764 Sextuplets, some liveborn  Z3769 Other multiple births, some liveborn  Z377 Other multiple births, all stillborn  Z379 Outcome of delivery, unspecified  O6010X0-9 Preterm labor w preterm delivery, unsp trimester  O6012X0-9 Preterm labor second tri w preterm delivery second tri  O6013X0-9 Preterm labor second tri w preterm delivery third tri  O6014X0-9 Preterm labor third tri w preterm delivery third tri  O6020X0-9 Term delivery with preterm labor, unspecified trimester  O6022X0-9 Term delivery with preterm labor, second trimester  O6023X0-9 Term delivery with preterm labor, third trimester |
|  | eProcedures.03 | 177184002 Normal delivery procedure  18540005 Suction of newborn  22633006 Vaginal delivery, medical personnel present  236973005 Delivery procedure/obstetric delivery  236994008 Placental delivery procedure  236996005 Abdominal uterine fundal massage  238248005 Umbilical cord clamping  408987002 Newborn care assessment  408989004 Newborn care management  409006000 Delivery care  409012005 Assess delivery care  423589000 Newborn continuous physical assessment  447214008 Ligation of umbilical cord of fetus  56620000 Delivery of placenta following delivery of infant outside of hospital  700000006 Vaginal delivery of fetus  8390008 Routine care of newborn  85403009 Delivery, medical personnel present |
| Preterm delivery | eSituation.09  eSituation.10  eSituation.11  eSituation.12 | O6010X0-9 Preterm labor w preterm delivery, unsp trimester  O6012X0-9 Preterm labor second tri w preterm delivery second tri  O6013X0-9 Preterm labor second tri w preterm delivery third tri  O6014X0-9 Preterm labor third tri w preterm delivery third tri |
| Multiple gestation delivery | eSituation.09  eSituation.10  eSituation.11  eSituation.12 | Z372 Twins, both liveborn  Z373 Twins, one liveborn and one stillborn  Z374 Twins, both stillborn  Z3750 Multiple births, unspecified, all liveborn  Z3751 Triplets, all liveborn  Z3752 Quadruplets, all liveborn  Z3753 Quintuplets, all liveborn  Z3754 Sextuplets, all liveborn  Z3759 Other multiple births, all liveborn  Z3760 Multiple births, unspecified, some liveborn  Z3761 Triplets, some liveborn  Z3762 Quadruplets, some liveborn  Z3763 Quintuplets, some liveborn  Z3764 Sextuplets, some liveborn  Z3769 Other multiple births, some liveborn  Z377 Other multiple births, all stillborn  O30001 Twin preg, unsp num plcnta & amnio sacs, first trimester  O30002 Twin preg, unsp num plcnta & amnio sacs, second trimester  O30003 Twin preg, unsp num plcnta & amnio sacs, third trimester  O30009 Twin pregnancy, unsp num plcnta & amnio sacs, unsp trimester  O30011 Twin pregnancy, monochorionic/monoamniotic, first trimester  O30012 Twin pregnancy, monochorionic/monoamniotic, second trimester  O30013 Twin pregnancy, monochorionic/monoamniotic, third trimester  O30019 Twin pregnancy, monochorionic/monoamniotic, unsp trimester  O30021 Conjoined twin pregnancy, first trimester  O30022 Conjoined twin pregnancy, second trimester  O30023 Conjoined twin pregnancy, third trimester  O30029 Conjoined twin pregnancy, unspecified trimester  O30031 Twin pregnancy, monochorionic/diamniotic, first trimester  O30032 Twin pregnancy, monochorionic/diamniotic, second trimester  O30033 Twin pregnancy, monochorionic/diamniotic, third trimester  O30039 Twin pregnancy, monochorionic/diamniotic, unsp trimester  O30041 Twin pregnancy, dichorionic/diamniotic, first trimester  O30042 Twin pregnancy, dichorionic/diamniotic, second trimester  O30043 Twin pregnancy, dichorionic/diamniotic, third trimester  O30049 Twin pregnancy, dichorionic/diamniotic, unsp trimester  O30091 Twin preg, unable to dtrm num plcnta & amnio sacs, first tri  O30092 Twin preg, unable to dtrm num plcnta & amnio sacs, 2nd tri  O30093 Twin preg, unable to dtrm num plcnta & amnio sacs, third tri  O30099 Twin preg, unable to dtrm num plcnta & amnio sacs, unsp tri  O30101 Triplet preg, unsp num plcnta & amnio sacs, first trimester  O30102 Triplet preg, unsp num plcnta & amnio sacs, second trimester  O30103 Triplet preg, unsp num plcnta & amnio sacs, third trimester  O30109 Triplet preg, unsp num plcnta & amnio sacs, unsp trimester  O30111 Triplet preg w two or more monochorionic fetuses, first tri  O30112 Triplet preg w two or more monochorionic fetuses, second tri  O30113 Triplet preg w two or more monochorionic fetuses, third tri  O30119 Triplet preg w two or more monochorionic fetuses, unsp tri  O30121 Triplet preg w two or more monoamnio fetuses, first tri  O30122 Triplet preg w two or more monoamnio fetuses, second tri  O30123 Triplet preg w two or more monoamnio fetuses, third tri  O30129 Triplet preg w two or more monoamnio fetuses, unsp trimester  O30131 Triplet pregnancy, trichorionic/triamniotic, first trimester  O30132 Triplet pregnancy, trichorionic/triamniotic, second trimester  O30133 Triplet pregnancy, trichorionic/triamniotic, third trimester  O30139 Triplet pregnancy, trichorionic/triamniotic, unspecified trimester  O30191 Trp preg, unable to dtrm num plcnta & amnio sacs, first tri  O30192 Trp preg, unable to dtrm num plcnta & amnio sacs, second tri  O30193 Trp preg, unable to dtrm num plcnta & amnio sacs, third tri  O30199 Trp preg, unable to dtrm num plcnta & amnio sacs, unsp tri  O30201 Quad preg, unsp num plcnta & amnio sacs, first trimester  O30202 Quad preg, unsp num plcnta & amnio sacs, second trimester  O30203 Quad preg, unsp num plcnta & amnio sacs, third trimester  O30209 Quad pregnancy, unsp num plcnta & amnio sacs, unsp trimester  O30211 Quad preg w two or more monochorionic fetuses, first tri  O30212 Quad preg w two or more monochorionic fetuses, second tri  O30213 Quad preg w two or more monochorionic fetuses, third tri  O30219 Quad preg w two or more monochorionic fetuses, unsp tri  O30221 Quad preg w two or more monoamnio fetuses, first trimester  O30222 Quad preg w two or more monoamnio fetuses, second trimester  O30223 Quad preg w two or more monoamnio fetuses, third trimester  O30229 Quad preg w two or more monoamnio fetuses, unsp trimester  O30231 Quadruplet pregnancy, quadrachorionic/quadra-amniotic, first trimester  O30232 Quadruplet pregnancy, quadrachorionic/quadra-amniotic, second trimester  O30233 Quadruplet pregnancy, quadrachorionic/quadra-amniotic, third trimester  O30239 Quadruplet pregnancy, quadrachorionic/quadra-amniotic, unspecified trimester  O30291 Quad preg, unable to dtrm num plcnta & amnio sacs, first tri  O30292 Quad preg, unable to dtrm num plcnta & amnio sacs, 2nd tri  O30293 Quad preg, unable to dtrm num plcnta & amnio sacs, third tri  O30299 Quad preg, unable to dtrm num plcnta & amnio sacs, unsp tri  O30801 Oth multiple gest, unsp num plcnta & amnio sacs, first tri  O30802 Oth multiple gest, unsp num plcnta & amnio sacs, second tri  O30803 Oth multiple gest, unsp num plcnta & amnio sacs, third tri  O30809 Oth multiple gest, unsp num plcnta & amnio sacs, unsp tri  O30811 Oth mult gest w two or more monochorionic fetuses, first tri  O30812 Oth mult gest w two or more monochorionic fetuses, 2nd tri  O30813 Oth mult gest w two or more monochorionic fetuses, third tri  O30819 Oth mult gest w two or more monochorionic fetuses, unsp tri  O30821 Oth multiple gest w two or more monoamnio fetuses, first tri  O30822 Oth mult gest w two or more monoamnio fetuses, second tri  O30823 Oth multiple gest w two or more monoamnio fetuses, third tri  O30829 Oth multiple gest w two or more monoamnio fetuses, unsp tri  O30831 Other specified multiple gestation, number of chorions and amnions are both equal to the number of fetuses, first trimester  O30832 Other specified multiple gestation, number of chorions and amnions are both equal to the number of fetuses, second trimester  O30833 Other specified multiple gestation, number of chorions and amnions are both equal to the number of fetuses, third trimester  O30839 Other specified multiple gestation, number of chorions and amnions are both equal to the number of fetuses, unspecified trimester  O30891 Oth mult gest, unab to dtrm num plcnta & amnio sacs, 1st tri  O30892 Oth mult gest, unab to dtrm num plcnta & amnio sacs, 2nd tri  O30893 Oth mult gest, unab to dtrm num plcnta & amnio sacs, 3rd tri  O30899 Oth mult gest,unab to dtrm num plcnta & amnio sacs, unsp tri  O3090 Multiple gestation, unspecified, unspecified trimester  O3091 Multiple gestation, unspecified, first trimester  O3092 Multiple gestation, unspecified, second trimester  O3093 Multiple gestation, unspecified, third trimester |
| Cardiac arrest (any etiology) | eSituation.09  eSituation.10  eSituation.11  eSituation.12 | I462 Cardiac arrest due to underlying cardiac condition  I468 Cardiac arrest due to other underlying condition  I469 Cardiac arrest, cause unspecified  I4901 Ventricular fibrillation  I4902 Ventricular flutter  I97120 Postprocedural cardiac arrest following cardiac surgery  I97121 Postprocedural cardiac arrest following other surgery  I97710 Intraoperative cardiac arrest during cardiac surgery  I97711 Intraoperative cardiac arrest during other surgery  O29111 Cardiac arrest due to anesthesia during pregnancy, first trimester  O29112 Cardiac arrest due to anesthesia during pregnancy, second trimester  O29113 Cardiac arrest due to anesthesia during pregnancy, third trimester  O29119 Cardiac arrest due to anesthesia during pregnancy, unspecified trimester  O0881 Cardiac arrest following an ectopic and molar pregnancy  O0486 Cardiac arrest following (induced) termination of pregnancy  O0736 Cardiac arrest following failed attempted termination of pregnancy  O0336 Cardiac arrest following incomplete spontaneous abortion  O0386 Cardiac arrest following complete or unspecified spontaneous abortion |
|  | eArrest.01 | 3001003 Yes, before EMS arrival  3001005 Yes, after EMS arrival |
|  | eProcedures.03 | 69779005 Cardiac resuscitation  89666000 CPR - Cardiopulmonary resuscitation  233169004 Automatic defibrillator procedure  308842001 Direct current defibrillation  426220008 External ventricular defibrillation  428805003 Advanced cardiopulmonary resuscitation  428909008 Biphasic defibrillation procedure  429283006 Mechanically assisted chest compression  429500007 Monophasic defibrillation  439569004 Resuscitation  439868004 Direct current ventricular defibrillation  441893003 Active compression decompression cardiopulmonary resuscitation with use of inspiratory impedance threshold device  431414003 Management of external defibrillation |

Abbreviations: NEMSIS, National Emergency Medical Services Information System.

*International Classification of Diseases, Tenth Revision, Clinical Modification (ICD-10-CM) codes or SNOMED CT concept ID codes (for procedures).
