# Supplementary material for: Frequency and severity of prehospital obstetric events encountered by emergency medical services in the United States
Source: BMC Pregnancy Childbirth. 2021 Sep 24;21:655. doi: 10.1186/s12884-021-04129-1 (PMC8464145; doi:10.1186/s12884-021-04129-1)
Supplement: Supplementary file 3 — Additional file 3: Table S3. Scoring for the modified early obstetric warning system (MEOWS). [file 12884_2021_4129_MOESM3_ESM.docx]

**Table S3**. Scoring for the modified early obstetric warning system (MEOWS).

| **MEOWS Component*** | **Red Alert** | **Yellow Alert** |
| --- | --- | --- |
| Temperature**^†^**, ºC | <35 or >38 | 35-36 |
| Systolic blood pressure, mmHg | <90 or >160 | 90-100 or 150-160 |
| Diastolic blood pressure**^†^**, mmHg | >100 | 90-100 |
| Heart rate, beats per minute | <40 or >120 | 40-50 or 100-120 |
| Respiratory rate, breaths per minute | <10 or >30 | 21-30 |
| Oxygen saturation, % | <95 | - |
| Pain score (0-10) | - | 8-10 |
| Neurological response | Unresponsive or responsive to pain | Responsive to voice |

*One red or two yellow alert conditions indicates that a patient requires evaluation.

^†^Not available in the dataset and not included in analysis.
